# PTH: Redefining Reference Ranges in a Healthy Population—The Role of Interfering Factors and the Type of Laboratory Assay

**DOI:** 10.1155/2020/1053719

**Published:** 2020-02-21

**Authors:** Simona Censi, Maurizio Iacobone, Stefano Simmini, Jacopo Manso, Giulio Franceschet, Mario Plebani, Anna Chiara Frigo, Martina Zaninotto, Francesca Torresan, Giustina De Silvestro, Carla Scaroni, Caterina Mian, Valentina Camozzi

**Affiliations:** ^1^Endocrinology Unit, Department of Medicine (DIMED), University of Padua, Padua, Italy; ^2^Endocrine Surgery Unit, Department of Surgery, Oncology and Gastroenterology (DiSCOG), University of Padua, Padua, Italy; ^3^Laboratory Medicine, Department of Medicine (DIMED), University of Padua, Padua, Italy; ^4^Department of Cardiac, Thoracic and Vascular Sciences, Biostatistics, Epidemiology and Public Health Unit, University of Padua, Padua, Italy; ^5^Department of Transfusion Medicine, Padua University Hospital, Padova, Italy

## Abstract

*Introduction*. Parathyroid hormone (PTH) is a linear peptide constituted by 84 amino acids and active in its 1–84 form, but a wide range of PTH forms produced by its post-transcriptional modifications are present in blood. Many assays with different specificities are commercially available. The aim of our study was to compare a 2^nd^ and 3^rd^ generation in healthy population in order to better define the reference range in the healthy population residing in our region. *Materials and Methods*. 108 subjects (53 females and 55 males) referring to the transfusion donor were enrolled in the study centre in April 2016 and underwent PTH levels measurements with a 3^rd^ generation kit (chemiluminescent immunoassay DiaSorin Liaison) and with a 2^nd^ generation kit (immunoradiometric assay Total Intact PTH Assay (Coated Tube), Scantibodies). Also calcium, phosphate, creatinine, and 25OHD3 were measured. A questionnaire on lifestyle and dietary habits was obtained.

**Results:**

The median PTH values obtained with the 2^nd^ generation assay and the whole 3^rd^ generation assay were 20.26 pg/ml and 23.11 pg/ml, respectively. Bland–Altman method showed substantial concordance between the two PTH assays, although with an overestimation of the 3^rd^ generation method over the 2^nd^ generation method. There was no correlation between 3^rd^ generation PTH and 25OHD3 and creatinine. Calcium was negatively correlated with PTH only when measured with 3^rd^ generation kit.

**Conclusions:**

On the basis of our data, obtained from healthy subjects, we can conclude that the reference range used by our laboratory was too narrow and was necessary to reestablish normal ranges according to our population. This is useful to avoid hyperparathyroidism misdiagnosis.

## 1. Introduction

Parathyroid hormone (PTH) is a linear peptide constituted by 84 amino acids and released by parathyroid glands. The PTH present in the circulation is very assorted, being present also PTH fragments, depending from post-transcriptional modifications occurring in parathyroid cells and in other tissues, mainly in the kidneys and in the liver [1]. As a consequence, there is a “pool” of PTH peptides, also in healthy people, but, in particular, in many clinical conditions. This phenomenon is particularly evident in patients with end-stage renal disease, where carboxy terminal PTH fragments are more abundant than the intact 1–84 PTH form, being half-life of the former (1-2 hours) and very longer than the latter (5–10 minutes) [1]. But the biological activity of this hormone is dependent from his intact form, particularly from its first N-terminal amino acids.

First generation assays consisted of competitive radioimmunoassay based on the use of polyclonal antibodies raised against PTH extracted from bovine or porcine parathyroid glands in animal models. This method had interference with the specificity of the antibodies that mainly recognized carboxyl fragments, the PTH form predominant in the gland extracts used for immunization [2]. Afterwards, it was clarified that the N-terminal 1–34 PTH fragment is an isoform with the same biological activity of its complete 1–84 form [3], and thus, second-generation assays were developed, nowadays the most used worldwide. These second-generation methods consist in a noncompetitive assessment, “Sandwich” immunoassays, based on the use of two monoclonal antibodies, one directed against the N-terminal (aa 13–24) and one directed against the carboxyl amino-terminal (aa 39–84). Initially, these assays were thought to recognize the entire (1–84) PTH molecule, and they were considered the gold standard for PTH dosage [4]. Subsequently, it was demonstrated that this method is unable to distinguish between the intact (1–84) form from the other fragments without the very first amino acids (in particular the more abundant 7–84), devoid of any biological activity. This occurs because the antibody used has a higher affinity for amino acids around 20–25 and not for the first 4 amino acids, recognized by antibodies of the last generation [5]. Afterwards, assays with this specificity and commercially available were developed (third generation immunoassays) [6]. But also this technique has its pitfalls: these types of assays are not able to distinguish the biologically active molecule from a post-translational modified form with a phosphorylated serine (aa 17) known as nontruncated amino-terminal (NT-N) parathyroid hormone (NT-N PTH) [7]. NT-N PTH represents 10% of total PTH values and up to 15% in patients with end-stage renal disease or a severe hyperparathyroidism or parathyroid cancers [7]. Also within the commercial kit of the same generation, it is difficult to obtain comparable results, owing to different calibrations, matrix effect, and antibody avidity [8]; to obtain comparable results, it is fundamental to undergo a standardization, within healthy and affected (especially hemodialized and patients with primary hyperparathyroidism) subjects. Even more problematic is to compare assays of different generations, since nowadays lots of laboratories still use second-generation immunoassays.

In the literature, studies comparing the assays from the two generations in a healthy population are not available, and they would be useful to define the normal range for these assays and to understand their comparability.

The aim of our study was the assessment of the reference range for PTH values in a population of healthy blood donors, with the comparison of PTH values obtained with the second and third generation kits and the identification of the biological factors able to interfere with PTH values obtained with the two assays.

## 2. Materials and Methods

### 2.1. Patients

114 people (56 males and 58 females, between 18 and 68 years) referring to the transfusion donor centre in April 2016 were enrolled in the study. The study was performed in accordance with the guidelines proposed in the Declaration of Helsinki, and all patients gave their written consent to the use of their blood for research purposes. The following exclusion criteria were considered: a history of acute or chronic diseases affecting bone, medical treatment interfering with skeletal metabolism in the previous 6 months, heavy smoking (more than 10 cigarettes/day), alcohol intake of more than 3 alcohol units/day, pregnancy, menopause within the previous 5 years, a personal or family history of pathological osteoporotic fractures, and extreme (very high or very low) values of 25OHD3. 5 females were excluded because they were using cholecalciferol supplement, and 1 male was excluded because of its vitamin D status, represented by circulating calcifediol level (25OHD3) was very higher than the other people included in the study (192 nmol/L). So, 108 subjects were finally included in the study (55 males and 53 females). All 108 subjects were assayed with the 3^rd^ generation kit, while it was possible to use the 2^nd^ generation kit in a maximum of 78 subjects, selected from the total population enrolled. Blood donors underwent a survey to collect individual lifestyle data, such as age, sex, nationality, solar light exposure (</≥30 minutes a day), physical exercise (yes/no), food calcium intake by a frequency validated questionnaire of dairy products, seafood, calcium-rich water consumption [9], reproductive state (years since the menopause, number of pregnancies, and miscarriages), medical and pharmacological history with particular attention to diseases and therapies interfering with bone metabolism (these blood donors were excluded from the study), and smoke habit. As regards nutritional calcium intake, a score <7 points was considered insufficient. Moreover, anthropometric characteristics were collected (weight, height, hip and waist circumference, and blood pressure). An 8.5 ml blood sample was collected from each patient.

### 2.2. Laboratory Assays

The third-generation PTH assay employed was a chemiluminescent immunoassay (CLIA) DiaSorin Liaison (Stillwater, USA) (reference range: 4.6–26.8 pg/ml) while the second-generation kit was an immunoradiometric assay (IRMA), Total Intact PTH Assay (Coated Tube), Scantibodies (Santee, USA) (reference range:10–57 pg/ml). 25OHD3 was measured with a CLIA assay (DiaSorin, Stillwater, USA) (reference range: 75–250 nmol/L). Calcium (reference range: 2.10–2.55 mmol/l), phosphate (reference range: 0.87–1.45 mmol/L), and creatinine (reference range in male subjects 59–104 *μ*mol/L, reference range in female subjects: 45–84 *μ*mol/L) were measured using an enzymatic assay (Cobas 8000, Roche-Hitachi, GmbH, Mannheim, Germany).

### 2.3. Statistical Analysis

The Kolmogorov–Smirnov test was used to test the normal distribution of the variables. When a normal distribution was found, mean values and standard deviations were employed. When the distribution was not normal, the median values and interquartile ranges were used. Categorical variables (smoking habit and physical exercise) or quantitative categorized (sun exposure, calcium intake, dichotomized 25OHD, and 2^nd^ and 3^rd^ generation assays) were analysed using chi-square or Fisher exact test. The eventual presence of linear correlation between variables (BMI, age, hip and waist circumference, calcium, phosphate, 25OHD3, and creatinine) and 25OHD3 and PTH measured with the 2 kits were analysed using the Spearman coefficient. The Bland–Altman plot was used to analyse the agreement between the two PTH methods. The concordance between the two assays was evaluated with Lin's concordance correlation coefficient, with a 95% confidence interval (IC) estimated with the Bootstrap method, carrying out 2000 resamples. A *p* value <0.05 was considered statistically significant.

## 3. Results

Patients' characteristics are summarized in Tables 1 and 2. In the population enrolled, 16 subjects were smokers (less than 10 cigarettes/day). In Table 3, mean values with relative standard deviation (SD) for calcium, phosphate, creatinine, 25OHD3, and PTH with the 2^nd^ and 3^rd^ generation assays in the entire population and in the population divided based on 25OHD3 levels are reported. The median PTH value obtained with the intact PTH assay (2^nd^ generation assay) and the whole PTH assay (3^rd^ generation assay) where 16.0 pg/ml (reference range: 4.6–26.8 pg/ml) and 20.9 pg/ml (reference range: 10–57 pg/ml), respectively. We found 2/78 (2.6%) cases of elevated PTH with the 2^nd^ generation assay (being elevated values in these 2 patients 65 and 69 pg/ml) and 27/108 (25%) (range of elevated values: 28–70 pg/ml), with the third generation assay. The 2 patients that had high PTH levels with “intact” assay had elevated PTH levels also with the 3^rd^ generation assay.

In the whole series, only 3/108 (2.7%) were found to have mildly elevated calcium levels, and none of these patients had elevated PTH values.

Analysing the association between solar exposition (</≥30 minutes/day) using the cutoff of 50 nmol/L (limit between deficiency and insufficiency) and the cutoff of 75 nmol/L (limit between insufficiency and sufficiency) for 25OHD3, we did not find any correlation between the two parameters. Moreover, 25OHD3 status was not associated with age, sex, and BMI in our series. There was no association between the 3^rd^ generation abnormal PTH and 25OHD3 deficiency and insufficiency (Table 4). As regards the nutritional calcium intake and creatinine, there was no association with an elevated PTH, obtained with the 3^rd^ generation kit. It was not possible to analyse the association of 25OHD3, nutritional calcium intake, and creatinine and an elevated PTH measured with the 2^nd^ generation assay because subjects with a PTH found over the normality range with this kit were only 2/78, so the numerosity was not enough for a statistical analysis. On the contrary, there was a correlation between sex and PTH values obtained with both the kits: PTH values were significantly higher in males both with the 3^rd^ generation kit (females: median value of 19.5 pg/ml, range: 10.4–9.4 pg/ml; males: median of 21.4 pg/ml, range: 8.1–70 pg/ml *p*=0.0454) and the 2^nd^ generation assay (females: median value of 13 pg/ml, range: 7.0–37.0 pg/ml and males: median value of 19.5 pg/ml, range: 7.0–69.0 pg/ml, *p*=0.0143) (Table 5).

**Figure 1:**
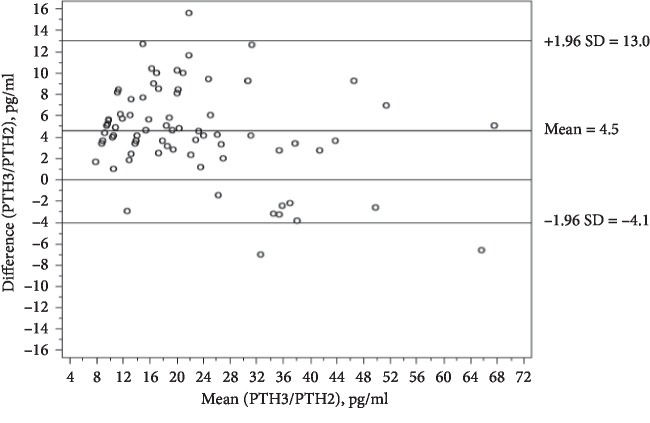
Bland–Altman plot analysing the agreement between the two PTH methods.

Age was found positively correlated with PTH when measured with both the kits; BMI is positively correlated with PTH when measured with the 2^nd^ generation kit; hip and waist circumferences are negatively correlated with 25OHD3 and positively with PTH values measured with 2^nd^ generation assay, while only waist circumference is positively correlated with PTH when measured with the 3^rd^ generation kit. Calcium levels are negatively correlated with PTH only when measured with the 3^rd^ generation kit, while phosphate is positively correlated only with 25OHD3. 25OHD3 is not correlated with PTH values, measured with both the assays. Creatinine is correlated only with 25OHD3, negatively. When the concordance between the two assays was analysed, the Bland–Altman method showed a mean error of 4.5 pg/ml, with an overestimation of the 3^rd^ generation method over the 2^nd^ generation method. Lin's concordance correlation coefficient was of 0.8917, with a 95% confidence interval of 0.8296 and 0.9387 (Figure 1).

## 4. Discussion

The study was inspired by the increasing number of asymptomatic patients with normal calcium concentrations referring to endocrinological consultation for increased PTH levels, when measured by our institutional laboratory. The problematic has emerged since the laboratory switched from an “intact” PTH second-generation assay kit to a “whole” third generation assay. PTH often resulted higher when obtained by our laboratory, when compared with laboratories that still used kits owning the previous generation. As a consequence, we wondered if the issue was due to the new assay kit used. Comparing PTH values obtained with different generation assays could be problematic. Tan and colleagues compared the third generation Roche Cobas Assay with four second-generation assays (Siemens ADVIA Centaur, OCD VITROS, Beckman Access 2, Abbott ARCHITECT, “intact” assays) in patients with renal disease. They concluded that Elycsys PTH (1–84) has a comparable precision and good correlation with “intact” assays; “intact” assays correlated well among each other but showed discrepancy with increasing PTH concentrations [10]. Another study compared a third-generation assay (Liaison, DiaSorin) with a second-generation assay (ELSA-PTH, Cis biomedical) in a population of dialyzed patients, and also, in this study, the two kits showed a good correlation, independently from age, sex, BMI, and blood pressure, but also, in this study, differences between the two assays increased with the rise of PTH values [11]. The aim of our study is to compare the two generations of PTH assays in healthy subjects, indeed this data is still not available in the literature.

So, a population of healthy subjects, with a personal and drug history negative for possible interference with bone metabolism, was enrolled. Then, the results on PTH values obtained with the two kits were compared. We chose to select our subjects among blood donors to have a healthy population, but many limitations remained. The limitations concerned the scarce representation of middle-aged people (30–40 years), having had this category of subjects a reduced availability to participate in the study and complete the questionnaire. Another issue is the difficulty in obtaining an objective quantification of sun exposure and calcium nutritional intake. Moreover, among the 108 subjects enrolled, only 7 (6.5%) had levels of vitamin D sufficiency (≥75 nmol/L), 40/108 (37%) had insufficient levels (between 50 and 75 nmol/L), and 61/108 (56.6%) even had deficient levels (<50 nmol/L). This result is not surprisingly since the recruitment was done at the beginning of springtime, correponding with the nadir of vitamin D status. This data could have affected the values of PTH found [12].

Older age and male sex were associated with higher PTH levels, maybe owing to a different sensitivity of parathyroid glands for serum calcium levels. Older age was found related to higher PTH values also in other studies [13], while gender and PTH association has obtained contrasting results in the literature, maybe owing to cultural issues and thus sun exposure [13]. It is possible that the PTH “resistance” develops with age and that a different PTH reference range should be adopted based on patients' age [14]. Moreover, also an impairment of renal function related with aging can contribute to this phenomenon. Thus, it is difficult to establish the ranges based on age, considering also other possible interfering factors that can play a role, from sun exposure, ethnicity, to menopause age [14, 15]. Based on these data, we can only say that the clinician should pay more attention to a slightly PTH elevation found in a young subject then to the same value documented in an elder.

Dietary habits and sun exposure did not influence PTH and 25OHD3 levels in our series, maybe owing to the daily variability of these parameters, and because of the difficulties related to its objective assessment.

No association was found between creatinine levels and PTH values, with both the assays. This result is probably due to the selection criteria including a population in good health.

Although the population considered was healthy, we found 2.6% of cases of elevated PTH with the 2^nd^ generation assay and up to 25% with the 3^rd^ generation assay, and all these patients had normal calcium levels. So, there is an imbalance in subjects with elevated PTH values with the two assays. The statistical analysis demonstrates a good concordance between the two assays, but indeed, there is an overestimation of the PTH values found with the 3^rd^ generation assay, and this discrepancy is more evident when low values (within the normal range) are considered. Maybe, for low PTH levels, 3^rd^ generation assay undergoes some and still unknown interference. This discrepancy would confirm doubts about the interchangeability between the two generations, and it is quite surprising to find higher PTH values with an assay that should be more specific for the whole PTH and should not recognize its fragments. The overestimation we obtained with the 3^rd^ generation assay is tolerable for low values but should not be acceptable for borderline values, situation in which a PTH overestimation should prompt mistaken clinical choices. A diagnosis of normocalcemic primary hyperparathyroidism can be made when the subject presents consistently elevated PTH concentrations in presence of normal calcium levels, after the exclusion of any cause of secondary PTH elevation (renal diseases, vitamin D insufficiency, etc) [15]. A narrow reference range can prompt the clinician to mistaken diagnosis and possibly to useless, time-consuming, and expensive diagnostic workout. Our laboratory adopted the normal range proposed by the literature obtained from an analogue Caucasian population (4.6–26.8 pg/ml) [16]. Based on our study, the normal range used in our laboratory was too narrow, with a highest value that should be raised to avoid a misdiagnosis of hyperparathyroidism, especially normocalcemic hyperparathyroidism, in healthy subjects. So, we suggest the normal laboratory range to be revised on the basis of these results, obtained in a population of healthy subjects residing in our region. However, the natural history of normocalcemic hyperparathyroidism is still unknown. Many patients become hypercalcemic and have evidence of organ damages, such as an impaired bone mineral density.

Another limit of the study could have been represented by the fact that our series included lot of people with insufficient vitamin D values, with possible consequently higher PTH values. By the way, this is not necessarily true in the healthy population. The Institute of Medicine (IOM) suggests there is no or only few evidence that, in the general population, a vitamin D level higher than 50 nmol/L is beneficial [17, 18], with acceptable levels for physiological functions in a healthy subject between 30 and 40 nmol/L, with a maximum calcium absorption capacity between 20 and 50 nmol/L [19]. IOM suggests that Endocrine Society guidelines on the treatment and prevention of vitamin D deficiency are based on weak and misinterpreted studies [20–22]. In conclusion, given the absence in our series of very high PTH values (we chose to consider a presumed healthy population), the scarce numerosity of subjects studied and the absence of important data for the evaluation of phosphocalcium metabolism (such as albumin and urinary calcium concentration), we cannot sustain the superiority of one assay on the other. Moreover, another limit of our study was the relatively scarce numerosity of patients in which we could obtain a PTH measurement with the 2^nd^ generation assay (78 patients), since kit availability was limited. The patients were randomly chosen, but we should consider the possibility that this could have influenced the results obtained. By the way, since the correlation between the two assays was good, this possibility is unlikely.

Further studies are needed, based on a series of patients possibly with ascertained sufficient vitamin D levels and with the availability of other factors that interfere on bone metabolism (like urinary calcium levels and albumin concentrations).

In conclusion, the purpose of our study was to place the emphasis on the importance of the assay and proper range used when evaluating PTH, in order to properly interpret the results, avoiding successive cost- and time-consuming laboratory and radiological tests.

## Figures and Tables

**Table 1:** Anthropometric characteristics of the enrolled population.

	All 108 (100%)	Females 53 (49.1%)	Males 55 (50.9%)
Age (years), median (range)	43.0 (18.0–68.0)	43.0 (18.0–59.0)	43.0 (20.0–68.0)
BMI (kg/m^2^) median (range)	24.1 (18.7–40.1)	23.1 (18.7–35.0)	25.3 (20.2–40.1)
Weight (kg) median (range)	72.0 (49.0–130.0)	63.0 (49.0–94.0)	80.0 (62.0–130.0)
Height (cm) median (range)	172.0 (150.0–192.0)	165.0 (150.0–175.0)	178.0 (160.0–192.0)
Waist circumference (cm) median (range)	85.5 (65.0–125.0)	77.0 (65.0–112.0)	90.0 (70.0–125.0)
Hip circumference (cm) median (range)	102.0 (86.0–130.0)	100.0 (86.0–130.0)	103.0 (93.0–120.0)
Median systolic blood pressure (mmHg) (range)	120.0 (95.0–185.0)	120.0 (95.0–140.0)	125.0 (100.0–185.0)
Median diastolic blood pressure (mmHg) (range)	80.0 (65.0–105.0)	80.0 (65.0–95.0)	80.0 (70.0–105.0)

**Table 2:** Food and behavioural habits of the enrolled population.

	All 108 (100%)	Females 53 (49.1%)	Males 55 (50.9%)
Physical exercise (number of patients)			
Yes	84 (100.0%)	42 (50.0%)	42 (50.0%)
No	24 (100.0%)	11 (45.8%)	13 (54.2%)
Sun exposure (number of patients)			
<30 minutes	35 (100.0%)	17 (48.6%)	18 (51.4%)
≥30 minutes	73 (100%)	36 (49.3%)	37 (50.7%)
Daily calcium intake (points mean value ± SD and median with range)	5.4 ± 2.4 5.0 (0.0–11.0)	5.2 ± 2.2 5.0 (1.0–11.0)	5.7 ± 2.5 6.0 (0.0–11.0)
Daily calcium intake (number of patients)			
<7 points	69 (100.0%)	37 (53.6%)	32 (46.4%)
Daily calcium intake (number of patients)			
≥7 points	39 (36.1%)	16 (41.0%)	23 (59.0%)

**Table 3:** Laboratory data categorized on the basis of 25OHD3 levels deficiency (≥50 nmol/L) or sufficiency (≥75 nmol/L), expressed in mean ± standard deviation and median values and the range of the values. Reference ranges: calcium: 2.10–2.55 nmol/L; creatinine: male subjects: 59–104 *μ*mol/L, female subjects: 45–84 *μ*mol/L; phosphate: 0.87–1.45 mmol/L; 3^rd^ generation PTH assay: 4.6–26.8 pg/ml; 2^nd^ generation PTH assay: 10–57 pg/ml.

	All patients 108 (100%)	25OHD3 < 50 nmol/L 61 (56.5%)	25OHD3 ≥ 50 nmol/L 47 (43.5%)	*p* value	25OHD3 < 75 nmol/L 101 (93.5%)	25OHD3 ≥ 75 nmol/L 7 (6.5%)	*p* value
Calcium (mean values ± SD) (mmol/L)	2.4 ± 0.1	2.4 ± 0.1	2.4 ± 0.1	0.69	2.4 ± 0.1	2.4 ± 0.1	0.64

Phosphate (mean values ± SD) (mmol/L)	1.1 ± 0.2	1.1 ± 0.2	1.1 ± 0.2	0.12	1.1 ± 0.2	1.3 ± 0.1	0.03

Creatinine (mean values ± SD) (*μ*mol/L)	77.4 ± 13.0	79.2 ± 14.1	74.9 ± 11.2	0.07	77.9 ± 13.1	70.4 ± 10.9	0.07

2^nd^ generation (median values and range) (pg/ml) PTH	16.0 (7.0–69.0)	17.5 (7.0–69.0)	14.0 (7.0–48.0)	0.09	16.0 (7.0–69.0)	14.0 (8.4–36.0)	0.28

3^rd^ generation (median values and range) (pg/ml) PTH	20.9 (8.1–70.0)	21.6 (11.0–70.0)	18.7 (8.1–54.9	0.19	20.7 (8.1–70.0)	22.0 (11.0–39.4)	0.96

**Table 4:** Blood donors divided according to the cutoffs of 25OHD3 levels, deficiency (≥50 nmol/L), and sufficiency (≥75 nmol/L) and according to PTH levels obtained with the third-generation assay (>26.8 pg/ml versus >4.6 and ≤26.8 pg/ml and <4.6 pg/ml).

	All patients 108 (100%)	PTH 3^rd^ generation >26.8 pg/m 28/114 (25.0%)	PTH 3^rd^ generation >4.6 and ≤26.8 pg/ml 86/114 (75.0%)	PTH 3^rd^ generation <4.6 pg/ml 0/114 (0%)	*p* value
25OHD3 < 50 nmol/L	61 (100)	16 (26%)	45 (74%)	0 (0%)	0.74
25OHD3 ≥ 50 nmol/L	47 (100)	11 (23%)	36 (77%)	0 (0%)	
25OHD3 ≥ 75 nmol/L	7 (100)	2 (29%)	5 (71%)	0 (0%)	0.82
25OHD3 < 75 nmol/L	101 (100)	25 (25%)	76 (75%)	0 (0%)	

**Table 5:** PTH values measured with 2^nd^ and 3^rd^ generation assay according to sex. Reference ranges: 3^rd^ generation PTH assay: 4.6–26.8 pg/ml; 2^nd^ generation PTH assay: 10–57 pg/ml.

	All patients 108 (100%)	PTH 3^rd^ generation (median and range) pg/ml	*p* value	All patients 75 (100%)	PTH 2^nd^ generation (median and range) pg/ml	*p* value
Females	53 (49.1%)	19.5 (10.4–9.4)	0.0454	37 (49.3%)	13.0 (7.0–37.0)	0.0143
Males	55 (50.9%)	21.4 (8.1–70.0)		38 (50.7%)	19.5 (7.0–69.0)	

## Data Availability

The biochemical data and questionnaires results used to support the findings of this study are available from the corresponding author upon request
